# Exotic grass invasion alters microsite conditions limiting woody recruitment potential in an Australian savanna

**DOI:** 10.1038/s41598-018-24704-5

**Published:** 2018-04-26

**Authors:** Samantha A. Setterfield, Peter J. Clifton, Lindsay B. Hutley, Natalie A. Rossiter-Rachor, Michael M. Douglas

**Affiliations:** 1 Faculty of Science, University of Western Australia, Crawley, Western Australia 6009 Australia; 2 Research Institute for the Environment and Livelihoods, National Environmental Science Programme (NESP), Charles Darwin University, Darwin, Northern Territory 0909 Australia

## Abstract

*Andropogon gayanus* Kunth. is a large African tussock grass invading Australia’s tropical savannas. Invasion results in more intense fires which increases the mortality rate of adult woody plants. Invasion may also affect community structure by altering the recruitment potential of woody plants. We investigated the effects of *A*. *gayanus* invasion on ground-level microclimate, and the carbon assimilation potential and recruitment potential of two *Eucalyptus* species. We compared microclimatic variables from the early wet-season and into the mid-dry season to coincide with the period of growth of *A*. *gayanus*. We assessed *Eucalyptus* recruitment by monitoring seedling establishment, growth and survival of experimentally sown seed, and estimating seedling density resulting from natural recruitment. *A*. *gayanus* invasion was associated with increased grass canopy height, biomass and cover. Following invasion, the understorey microclimate had significantly reduced levels of photon flux density, increased air temperatures and vapour pressure deficit. The conditions were less favourable for woody seedling with aboveground biomass of seedlings reduced by 26% in invaded plots. We estimated that invasion reduced daily carbon assimilation of woody seedlings by ~30% and reduced survivorship of *Eucalyptus* seedlings. Therefore, *A*. *gayanus* invasion reduces recruitment potential, contributing to the transformation of savanna to a grassland ecosystem.

## Introduction

Invasive alien grasses threaten the structure and function of many of the world’s tropical savanna ecosystems^1–4^. Ecological impacts include change in the plant community composition and structure, changes in nutrient cycling and altered fire regimes^4–8^. Invasive plants can affect regeneration of native plants by suppressing seed germination and altering the ground layer so that seedling establishment and survival are reduced^9–11^. The alien C4 perennial grass *Andropogon gayanus* (gamba grass) is invading large areas of the tropical savanna ecosystems of northern Australia^2,12^. *A*. *gayanus* has higher rates of stomatal conductance, assimilation and water use; a longer daily assimilation period and growing season; and higher photosynthetic nitrogen use efficiency than the native grass species^13^. Coupled with fire tolerance in a highly flammable ecosystem, these factors confer a significant competitive advantage over native grass species. The rapid invasion and high impact of this species is reflected by its status as one of Australia’s 32 Weeds of National Significance^14^.

*A*. *gayanus* differs structurally from Australia’s native savanna grass species as it forms taller tussocks (typically 2–5 m *c*.*f*. 0.5–3 m for native grasses) with a larger basal area (up to 70 cm diameter *c*.*f*. up to 20 cm for native grasses). Early invasion is characterised by a change in the structure of the ground layer as *A*. *gayanus* tussocks displace the native species^15^. As invasion proceeds, a near complete replacement of the native understorey species occurs, resulting in significantly increased fuel loads and fires that are up to five times more intense than native grass fires^8,16^. This can ultimately lead to a reduction in the cover and density of the overstorey trees^12,17^. This is a significant change in this tropical savanna ecosystem which is characterised by frequent fire, generally occurring at intervals of 1–3 years^18^.

In addition to altering the community structure via increased tree and shrub mortality, *A*. *gayanus* invasion may alter the recruitment potential of woody species. Recruitment of woody seedlings depends on the supply of water, light and nutrients relative to the demand for these resources from herbaceous competitors^19^. Grass species vary in their level of resource use and changes in grass composition can affect the availability of resources to woody seedlings^20,21^. Changes in resource availability and stand structure affect microclimatic variables at ground level, such as the solar radiation regime (both quantity and spectral composition of radiation), soil and air temperature, humidity and wind dynamics^22–24^. Ground-level microclimate has a strong influence on seedling recruitment and changes caused by exotic species have affected the germination, growth and survival of woody seedlings in a range of ecosystems^20,25–28^.

The aim of this study was to investigate whether *A*. *gayanus* invasion affects: (1) ground-level microclimate; and (2) the establishment, growth and survival of woody seedlings. Our study site contained ‘invaded’ plots, which were at the early stage of invasion with a dense *A*. *gayanus* understorey but with a relatively intact native woody species overstorey^12,15^, and ‘native grass’ plots which did not contain *A*. *gayanus* in the grass layer. We hypothesised that dense *A*. *gayanus* invasion would alter both soil and near-ground air temperature and humidity and thus vapour pressure deficit (VPD), reduce incident radiation at ground level and reduce soil moisture during the late wet season when the grass canopy reaches maturity. If changes in microclimate are detected, we would expect to also detect a reduction in recruitment, growth rate, and survival of *Eucalyptus* seedlings in invaded and native grass dominated plots.

## Results

### Vegetation structure

The invasion by *A*. *gayanus* resulted in marked change in the structure of the savanna ground layer, particular by the mid-wet season. In November, at the beginning of the wet season, when ground-layer species are germinating and establishing prior to their main growth period^29,30^, the grass cover (10–15%) did not vary significantly between native and invaded savanna (Table 1). By February, grass cover was significantly higher in the invaded plots (30%) compared to the native grass plots (15%) and this continued throughout the wet season and into the mid-dry season (Table 1). The grass layer was significantly taller in invaded plots throughout the wet season (Table 1) and by the mid-dry season (July), mean grass height was nearly an order of magnitude higher in the invaded plots (2.1 m compared to 0.39 m native grass; Table 1). The invaded plots had a significantly lower forb cover in February (~20% c.f. ~40% in native plots) and April (~9% c.f. ~25% in native plots), by which time forb species had begun to senesce and covered less than 5% of the quadrats by July (Table 1). The sites used in this study were in the early stage of invasion, defined by the alternative state in which the overstory remains intact but the native understory is significantly displaced by *A*. *gayanus*^9,15^.

**Table 1:** The mean (SE) of (a) projected grass cover, (b) mean grass height, (c) tree canopy cover and (d) forb cover measured in 15, 1 m^2^ plots in each of four sites dominated by native grasses (native) or *A*. *gayanus*.

	November	February	April	July
**(a) Grass cover (%)**				
Native	10.1 (1.6)	15.5 (11.3)	25.1 (13.2)	23.8 (1.7)
Invaded	14.6 (1.7)	30.3 (1.9)	45.5 (17.6)	31.4 (1.9)
*F value (df* = *1*, *3)*		10.4*	122.2***	101.1**
**(b) Grass height (m)**				
Native	0.22 (0.01)	0.29 (0.01)	0.4 (0.03)	0.13 (0.02)
Invaded	0.34 (0.17)	0.81 (0.04)	1.5 (0.07)	2.1 (0.11)
*F value (df* = *1*, *3)*	23*	54**	27.5*	30.7**
**(c) Forb cover (%)**				
Native	7.8 (1.7)	41.6 (1.9)	24.6 (1.3)	3.4 (0.8)
Invaded	3.1 (0.6)	19.3 (1.7)	9.2 (1.5)	1.2 (0.5)
*F value (df* = *1*, *3)*		22.14*	44.5***	

Measurements were made in wet season (November, and February, April) and mid-dry season (July). F values and degrees of freedom following two-factor mixed-model ANOVA are provided where there were statistically significant differences between plot types. Asterisks (*) represent significant differences between treatments (*P < 0.05, **P < 0.01, ***P < 0.001).

### Microclimate

**Figure 1:**
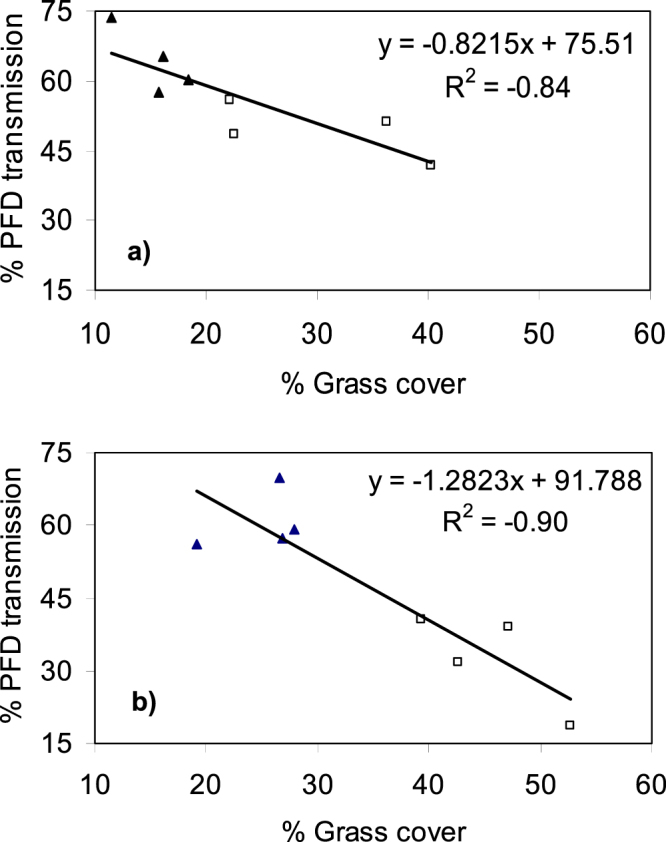
Relationship between grass cover and %PFD light transmitted at ground level in (**a**) February and (**b**) April. Symbols are mean values for native grass (solid triangle) and *A*. *gayanus* (open square) plots.

**Figure 2:**
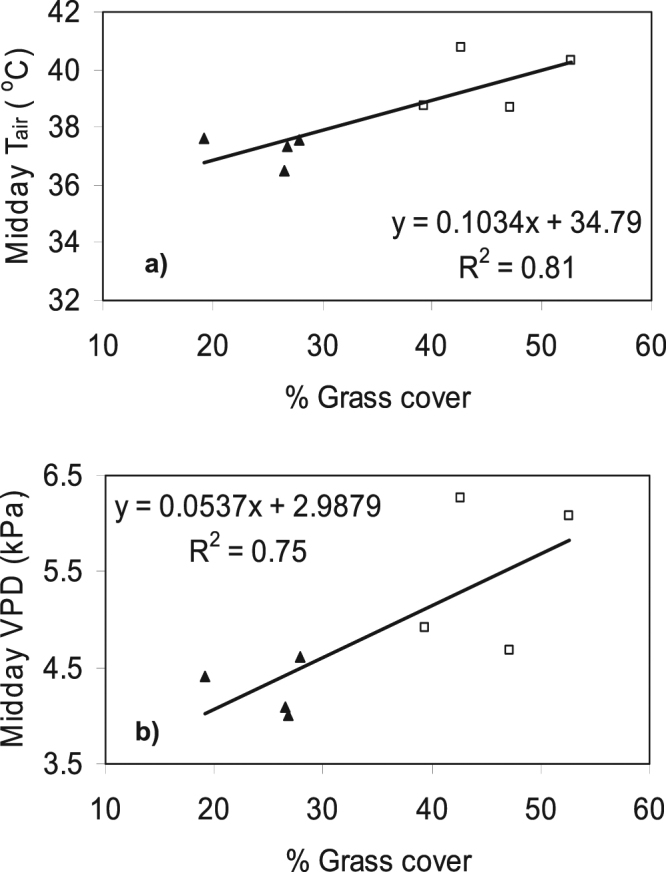
Relationship between grass cover and (**a**) midday air temperature and (**b**) midday VPD during April. Symbols are mean values for native grass (solid triangle) and *A*. *gayanus* (open square) plots.

**Figure 3:**
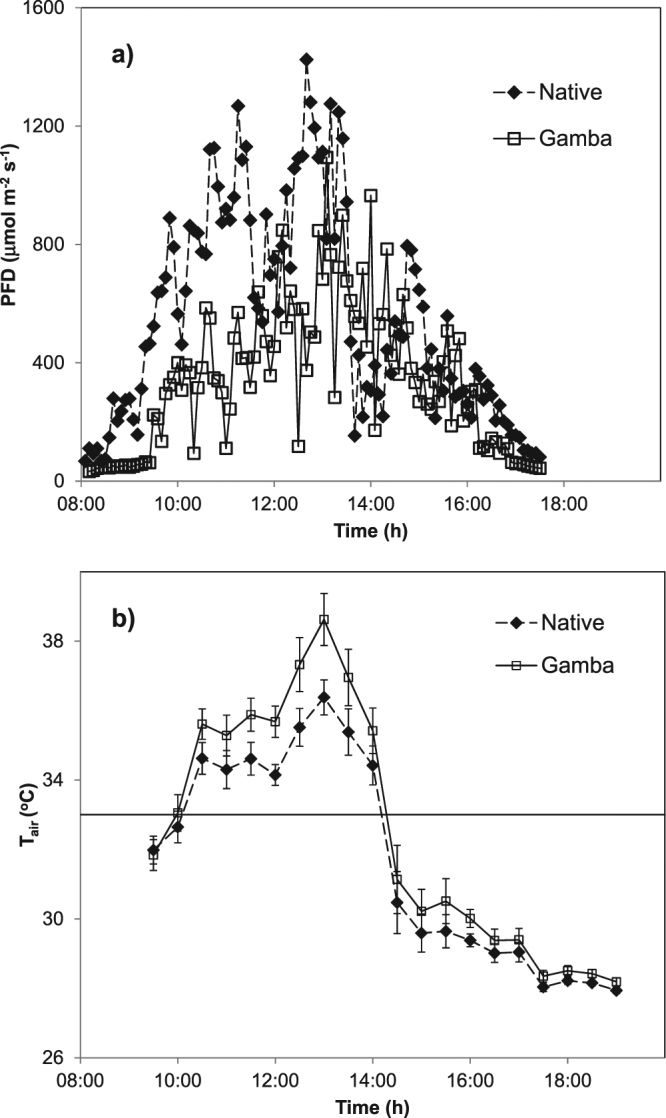
Typical diurnal patterns of (**a**) PFD and (**b**) T_air_ beneath gamba and native grass canopies. Data are composite means using diurnal observations from all sites. On panel (b), the solid line is the optimal leaf temperature for *Eucalyptus* seedling photosynthesis^28^.

The change in ground layer structure was correlated with change in a range of microclimate characteristics. As grass cover and height increased during the wet season, the percentage of light reaching ground level (photon flux density (PFD)) declined from 70% in November to 32.6% in April in the invaded plots, compared to a decline of 68.5% to 60.6% over the same time period in the native plots (Table 2); the decline was strongly correlated with grass cover (Fig. 1). PFD at ground level did not vary significantly between grass types in November but was significantly lower in the invaded plots compared to native grass plots in February and April (Table 2). The daytime mean (i.e. between 0900 and 1700 hr) air temperature within the grass layer (daytime mean T_air_) showed a similar pattern of change in light transmission. In November, there was no significant difference in daytime mean T_air_ between grass types, however from the mid-wet season to mid-dry season, the daytime mean T_air_ within invaded plots was 0.8 °C to 1.5 °C higher than native grass plots (Table 2). Similarly, the mean T_air_ at midday was significantly higher at all times of the year in invaded plots, increasing from a mean difference of 0.8 °C in November to 2.4 °C by April (Table 2). By July, midday temperatures had decreased by 1.9 °C. Mean T_air_ at midday correlated with grass cover (Fig. 2a). The nocturnal (i.e. 1900–0700 hr) T_air_ were not significantly different between invaded and native plots for most of the year with the only significant difference occurring in November (invaded plots 27.34 ± 0.2 °C c.f. gamba plots 26.94 ± 0.22 °C). We present the typical diurnal patterns of PFD and T_air_ for the two grass types in February, the peak growing season (Fig. 3). These patterns demonstrate the low radiation incident at 10 cm in invaded plots, with T_air_ elevated by a minimum of 1 °C between 11 to 2 pm, and a maximum difference of 2 °C at solar noon (1300hrs local time).

**Table 2:** The mean PFD transmission (%) to the ground surface; mean T_air_ (°C) for daytime, midday, and nocturnal periods as defined in the methods; mean VPD (kPa) for daytime and midday; mean T_soil_ (°C) for daytime and nocturnal periods; and daily mean θ_v_ (m^3^ m^−3^) in native and invaded plots in the wet season (November, February, April,) and mid-dry season (July).

Variable	Grass type	November	February	April	July
**PFD**	Native	68.5 (4.5)	64.1 (3.4)	60.6 (3.12)	36.2 (12.7)
	Invaded	70.0 (4.6)	49.3 (2.4)	32.6 (5.03)	32.0 (5.1)
	*F value (df* = *1*, *19)*		14.31**	23.25***	0.38
**T**_**air**_					
*Daytime*	Native	36.85 (0.12)	31.68 (0.13)	34.9 (0.18)	31.5 (0.29)
	Invaded	37.28 (0.15)	32.49 (0.19)	36.32 (0.32)	32.38 (0.31)
	*F value (df = 1*, *35)*		10.74*	17.47***	8.26**
*Midday*	Native	39.12 (0.24)	33.82 (0.15)	37.24 (0.32)	34.19 (0.35)
	Invaded	39.91 (0.26)	35.14 (0.26)	39.64 (0.53)	36.05 (0.05)
	*F value (df = 1*, *35)*	9.99**	30.15***	15.60***	15.87***
**VPD**					
*Daytime*	Native	3.60 (0.11)	1.32 (0.08)	3.33 (0.11)	3.45 (0.08)
	Invaded	3.90 (0.11)	1.44 (0.10)	3.96 (0.20)	3.87 (0.10)
	*F value (df = 1*, *11)*	40.0***		9.18*	17.85**
*Midday*	Native	4.64 (0.21)	1.97 (0.10)	4.28 (0.22)	4.27 (0.15)
	Invaded	5.10 (0.2)	2.28 (0.19)	5.48 (0.37)	5.14 (0.19)
	*F value (df = 1*, *11)*	15.07**		7.83*	19.90***
*Nocturnal*	Native	0.69 (0.06)	0.19 (0.02)	0.28 (0.02)	0.36 (0.03)
	Invaded	0.61 (0.06)	0.16 (0.01)	0.29 (0.02)	1.65 (0.08)
	*F value (df = 1*, *11)*				
**T**_**soil**_					
*Daytime*	Native	37.12 (0.53)	32.75 (0.19)	33.84 (0.49)	30.06 (0.49)
	Invaded	38.09 (0.56)	33.07 (0.35)	32.93 (0.42)	28.12 (1.04)
*Nocturnal*	Native	30.11 (0.19)	28.78 (0.08)	28.28 (0.20)	22.29 (0.43)
	Invaded	29.80 (0.22)	29.19 (0.18)	27.93 (0.12)	22.00 (0.43)
**θ**_***v***_	Native	0.044 (0.002)*	0.13(0.005)***	0.03 (0.002)	0.01 (0.001)
	Invaded *F value (df = 1*, *11)*	0.056 (0.002) 4.76	0.158 (0.006) 26.33	0.042 (0.002)	0.016 (0.004)

All values are means and SE in parenthesis. F values and degrees of freedom are provided where there were statistically significant differences between plot types. Asterisks (*) represent significant differences between treatments (*P < 0.05, **P < 0.01, ***P < 0.001).

The vapour pressure deficit (VPD) in the grass layer followed a similar trend to T_air_, with daytime VPD being significantly higher in invaded plots compared to native plots during November, April and July (Table 2). Midday VPD was also significantly higher in invaded plots for these 3 months. Nocturnal mean VPD was significantly lower in invaded plots during November but did not differ significantly for the remainder of the wet and dry season.

We estimated the potential woody seedling assimilation (Apot) based on previously established relationships between *Eucalyptus* seedling photosynthesis and light and temperature^31^. We used these functions to estimate the impacts of grass type on A_pot_ using diurnal PFD and T_air_ data for each grass type (Fig. 3). The daily integral of PFD beneath each canopy was 31% lower under gamba (11.4 ± 0.6 mol m^−2^ d^−1^) compared to native grass canopy (17.6 ± 2.6 mol m^−2^ d^−1^), which translated into a 27% reduction in A_pot_ (1-Way ANOVA, df = 1, F = 13.4, P < 0.01). At the end of the growing season, a similar and significant decline in biomass of *E*. *miniata* seedlings in invaded plots was also observed (24%, Table 3). Supra-optimal temperatures for photosynthesis beneath gamba canopies were evident. Figure 3b provides mean diurnal curves from the plot pairs with the optimal temperature for leaf-scale photosynthesis in *E*. *tetrodonta* seedlings (~33 °C^31^) also plotted. Beneath gamba canopies, supra-optimal temperatures occurred for 5 hours of the day and T_air_ was up to 6 °C above the optimal temperature for photosynthesis. In native sites, temperatures were 3 °C above the optimal. Estimating A_pot_ using these temperatures resulted in a lower A_pot_ (1-WayANOVA, df = 1, F = 8.51, P < 0.01) within invaded sites, although this effect on A_pot_ was not as large when driven by reduced radiation, with mean A_pot_ only 4% lower in invaded plots.

**Table 3:** Comparison of mean (±SE) biomass and morphological characteristics for *E*. *miniata* seedlings beneath native and gamba canopies at the end of the growing season.

Trait	Native grass	A. gayanus
Foliage area (cm^2^)	30.47 (1.79)	23.52 (1.37)
Specific leaf area (m^2^ kg^−1^)	87.54 (9.77)	106.08 (7.30)
Foliage: stem wt ratio	1.85 (0.10)	1.54 (0.11)
Internode length (mm)	29.26 (0.61)	29.52 (0.63)
Above-ground biomass (mg)	222.67 (14.62)*	164.89 (9.78)
Lignotuber biomass (mg)	22.67 (1.41)	21.11 (2.71)
Total biomass (mg)	245.3 (16.02)*	186.0 (12.49)*

Asterisks (*) denote significant differences (P < 0.05).

**Figure 4:**
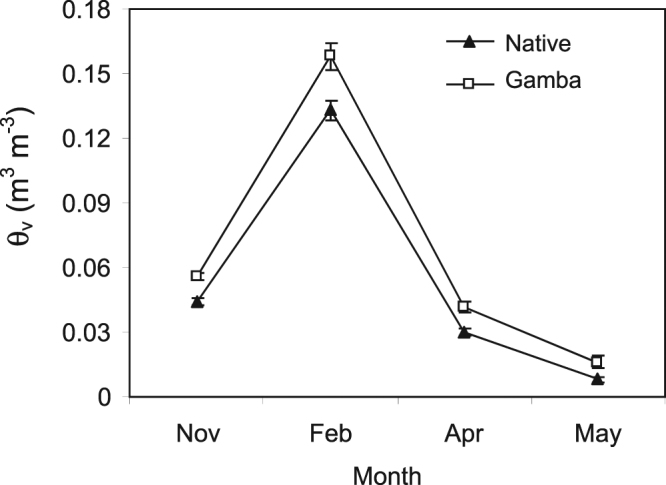
Seasonal changes in mean (±SE) surface soil moisture from November to April, late dry to the early dry season.

We found that volumetric soil moisture θ_v_ was consistently higher in invaded plots compared to native grass plots (Fig. 4) in November (F_1,11_ = 4.76, P < 0.05) and February (F_1,11_ = 26.33, P < 0.001). By April, measurements still showed a strong trend towards being higher in invaded plots (F_1,3_ = 9.83, P = 0.052), although the difference was reduced by May (Fig. 4).

### Seedling recruitment

There was no significant difference in seedling emergence between treatments for either *E*. *miniata* or *E*. *tetrodonta*. Mean emergence for *E*. *miniata* was higher in invaded plots with 44% of seeds emerging compared to 38% in native plots. Mean emergence of *E*. *tetrodonta* was 11% for both treatments. The ‘control’ (no seed augmentation) plots showed that no natural recruitment occurred in either plot type. Overall emergence of *E*. *tetrodonta* was significantly lower than *E*. *miniata* (F_1,2_ = 72.0, P < 0.05). Similarly, there was little variation in predation rates between treatments, except in December, with lower rates observed in invaded plots (F_1,2_ = 25.0, P < 0.05).

Despite differences in microclimate, there was no significant difference in height or number of leaves per seedling between grass treatments for either tree species. However, there was a significant difference in the above-ground biomass for *E*. *miniata* seedlings; mean above-ground biomass was 0.165 g dry weight in invaded plots compared to 0.223 g in native plots (F_1,14_ = 17.36, P < 0.001). Above-ground biomass for *E*. *miniata* in May was negatively associated with mean grass cover of monthly measurements recorded from December to May (r^2^ = −0.60, P < 0.01; df = 18). Despite differences in above-ground biomass, there was no significant difference in mean seedling lignotuber weight in invaded or native plots.

Seedling survival to the early dry season (May) varied significantly between grass treatment types (F_1,21_ = 4.60, P = 0.044) for both tree species with a reduction of 11% survival in invaded plots. Survival of *E*. *miniata* seedlings during the wet season was negatively correlated with mean grass cover over this period (r^2^ = −0.53, P < 0.05). Also, the number of *E*. *miniata* seedlings that died between late April and late May was negatively correlated with seedling above-ground biomass (r^2^ = −0.55, P < 0.05) although it was poorly correlated with lignotuber biomass. Natural *E*. *miniata* seedling recruitment was significantly lower in areas invaded by gamba (F_1,76_ = 5.21, P < 0.05) with a mean of 0.52 seedlings per tree in invaded plots compared to 2.14 seedlings per tree in native grass plots.

## Discussion

The maintenance of tree populations in savanna ecosystems requires suitable microenvironment for germination and growth of juveniles, either re-sprouts or seedlings^30,32^. The ground-layer microenvironment of savanna plots invaded by *A*. *gayanus* in our study site differed significantly from the microenvironment in native grass dominated plots despite its the relatively recent invasion^6^. Invaded plots had a dense grass cover that was almost twice that of native grass areas in the wet season, with an increase in grass layer height of up to 2 m in the late wet/early dry season. Consequently, there was a 30% reduction in radiation levels incident at ground level relative to uninvaded savanna. Reduced radiation load is likely to reduce the growth potential of many savanna woody seedlings. For example, *Eucalyptus* species growing in open forests and woodlands are light demanding species^33^, and for saplings of *E*. *tetrodonta* and *E*. *miniata*, light saturation for photosynthesis is typically high at approximately 1500 μmol m^−2^ s^−1 31^. Similarly, saplings of *Terminalia ferdinandiana* Exell., a common deciduous small tree of these open-forest savannas, is light saturated at 800 μmol m^−2^ s^−1^ during the wet season^34^. Elsewhere, invasive grasses have been shown to reduce biomass production of woody seedlings; Cabin *et al*.^25^ showed that *Pennisetum setaceums* invasion of Hawaiian tropical dry forests reduced ground level PFD by 55%, which caused a 31% reduction in the photosynthetic rates of woody seedlings compared to seedlings growing in uninvaded areas.

Reduced PFD within plant canopies generally results in reduced T_air_^23^, which is likely to minimise carbon loss via reduced rates of autotrophic respiration. However, in this study, the *A*. *gayanus* canopy was associated with an increase in T_air_ of 2.4 °C relative to adjacent native plots. This increase is likely to be due to a combination of factors, namely, increased grass density and biomass heat storage coupled with reduced within-canopy mixing of air. During the early wet season (November to January), when the grass canopies were not fully developed, the differences between invaded and native plots were minimal. As structural differences in grass canopies increased with growth through the wet season, there were changes in the diurnal patterns of T_air_. Daytime T_air_ became increasingly hotter in invaded plots compared to native plots, due to increased heat capture and storage by the developing *A*. *gayanus* grass cover and biomass. The increased T_air_ and VPD is likely to have significant negative impacts on growth potential of woody seedlings, by reducing their assimilation rates when the optimum temperature for photosynthesis is exceeded via increased autotrophic respiration^35^. The optimal temperature for assimilation for *E*. *tetrodonta* is ~33 °C (and assimilation declines rapidly above 35 °C^31,34^). Mean mid-day T_air_ within the grass layer of the invaded plots exceeded 35 °C for all measurement periods during the study and was particularly high during April (39.6 °C, Table 2). This suggests that for *Eucalyptus* seedlings, within-canopy T_air_ would be almost 10 °C above the optimal temperature for photosynthesis. During the growing season A_pot_ of *Eucalyptus* seedlings was 30% lower in invaded plots given the elevated temperatures and reduced transmitted radiation, similar to the observed fractional reduction in seedling biomass growing under gamba canopies (Table 3). Woody species of these savannas also regenerate via basal sprouts after disturbance such as insect damage and fire^32^, and the saplings would also experience altered microclimate conditions.

The impacts of VPD on seedling assimilation and stomatal conductance may be offset by the observed elevated surface soil θ_v_ within invaded plots, which was consistently higher compared to adjacent native plots. Higher soil moisture levels may have resulted from reduced evaporation rates due to the reduced radiation load and shaded soil surfaces and reduced turbulent mixing. Alternatively, this surface moisture may have resulted from hydraulic lift, with wetter sub-soils and drier surface soils providing the required water potential gradient. Hydraulic lift is not commonly reported in grass species, but C4 grasses such as *Aristida stricta* are capable of this phenomenon^36^. Elevated θ_v_ in *A*. *gayanus* plots occurred at the beginning of the wet season which could increase the likelihood of seedling emergence^37^ and may also provide additional nutrients in the top 10 cm of soil via increased rates of mineralisation^38,39^, potentially favouring *A*. *gayanus* growth given this species’ ability to assimilate both nitrate and ammonium as nitrogen sources^6^.

*A*. *gayanus* invasion did not negatively impact establishment of artificially sown *Eucalyptus* seed, but it did reduce survival of seedlings over the nine month dry-wet-dry seasonal monitoring period. The lack of impact on seedling establishment was not surprising given that seed fall and seedling recruitment occurs during the early wet season when differences in microclimate between grass types was minimal. In addition, there was no difference in seed predation rates between grass types. Ants are a major seed predator in the Australian savannas and a significant factor limiting the recruitment of woody species in northern Australian savannas^40^, however the ant community and composition is not significantly affected by *A*. *gayanus* despite the substantial changes in habitat structure resulting from invasion^15^. The major impact is therefore the significant reduction in *Eucalyptus* seedling biomass and survival, which was correlated with grass type and microclimate. Higher than optimal temperatures, VPD and light regime limited seedling photosynthesis beneath the canopy of *A*. *gayanus* reduces assimilation potential and lowered fitness^13^. This physiological impact has been detected in moderately invaded ( < 50% cover) sites^16^. Dense invasion can result in *A*. *gayanus* canopy approaching 100% cover^12^, which would further limit radiation transmission with increased temperatures and VPD likely.

In addition to unfavourable microclimatic effects, woody seedling recruitment in *A*. *gayanus* plots will be reduced over the longer-term by the shift in fire regime associated with invasion, with increased fire frequency and severity commonly observed in gamba patches^7^. Increased intensity and leaf scorch height increase heat damage to reproductive structures^41^, and also reduce flowering in years following fires^30^, possibly due to the diversion of resources to canopy maintenance at the expense of ovule development^42^, factors that would reduce potential seed fall, further limiting woody regeneration potential. Reduced seed input and seedling survival are likely to both be major contributors to the substantially lower number of naturally recruited seedlings in invaded plots compared to native grass plots.

In summary, this study has shown that even at a relatively early stage of invasion, the microclimate beneath *A*. *gayanus* canopies is characterised by high temperatures and light-limiting radiation. This altered microclimate will influence a range of process including seedling emergence, growth and survival. We have shown that the seedling growth and survival of a dominant woody overstorey species is reduced in the altered conditions. The density of established seedlings in invaded patches is significantly reduced when compared to the density in native grass patches. This is likely to be due to both reduced seed supply, due to the impacts of fire on seed production, and the direct impacts on seedling survivorship. These processes will result in savanna of low floristic diversity dominated by *A*. *gayanus* as opposed to the floristically rich natural savanna of this region that consists of four phenological guilds of woody species^43^ and a range of annual and perennial C4 grasses.

## Methods

### Site description

Our study was conducted at the Mary River National Park (12°38′S, 131°45′E), ~100 km southeast of Darwin, Northern Territory, Australia. Sites were located within open-forest savanna (*sensu* sensu^44^), with an average canopy cover of 50–60% and canopy height of 15–20 m^12^. Overstorey vegetation was dominated by *Eucalyptus miniata* A. Cunn ex Schauer and *E*. *tetrodonta* F. Muell. and the mid-storey consisting of deciduous and semi-deciduous small trees and shrubs. These two eucalypt species represent over 80% of the leaf area index and biomass of the NT’s mesic savanna^45^. The dominant native grasses are C4 grasses *Alloteropsis semialata* (R. Br.) A. Hitchc and *Eriachne triseta* Nees ex Steud^46^. This and similar savanna types occupy almost 200,000 km^2^ across northern Australia^47^. The climate is characterised by monsoonal rains and high humidity in the wet season (October-April) and virtually no rain with high rates of evaporation throughout the dry season (May-September). The mean annual rainfall for Mary River National Park is ~1570 mm^48^. Monthly mean maximum temperature increases significantly in September, peaking at approximately 37 °C in October/November, before stabilising to 33–34 °C for the remainder of the wet season, and reaching a minimum of 31 °C in the mid-dry season^48^. Experimental sites were on deeply weathered and partly laterised, late Tertiary sediments of the Koolpinyah surface and associated course sandy Quaternary alluvium. Soils were of the Kandosol order (after^49^ and varied between deep, gravel-free red kandosols and moderately deep red and red-yellow kandosols with some gravel.

### Sampling regime

Our study used a randomised block design at savanna sites described by Rossiter-Rachor *et al*.^50^. Four blocks were established (hereafter referred to as plot-pairs), with each plot-pair consisting of an area dominated by native grass (hereafter referred to as ‘native grass’ plots), and an adjacent (~50 m distant) *A*. *gayanus* dominated area (hereafter referred to as ‘invaded’ plots). Plot-pairs were located up to 600 m apart, and each plot was 50 × 50 m in size. All plot-pairs were burnt in May as part of the Park’s management burning program, prior to commencement of the experiment, and consequently there was very low (<10%) grass cover until the wet-season rains began in October, just prior to the initial measurements. Plot-pairs remained unburned for the duration of the data collection period.

### Vegetation and microclimate

We measured the vegetation structure and microclimate within experimental plots on four occasions during a full wet-dry cycle: November (pre-wet season), the following February (mid-wet), April (early-dry) and July (mid-dry season). Grass cover was characterised by randomly placing 15, 1 m^2^ quadrats within each plot (i.e. n = 30 quadrats per plot-pair) and estimating the percent cover of grasses (live and dead), forbs, re-shoots, litter and bare soil. Mean grass tussock height was also recorded based on five random measures per quadrat.

We assessed differences in near-surface microclimate by estimating canopy transmission of photon flux density (PFD) to the soil surface, and within-understorey canopy air temperature (T_air_) and relative humidity (RH%) to estimate within-understorey canopy vapour pressure deficit (VPD). Canopy transmission was estimated by comparing incoming PFD received at the soil surface beneath each grass canopy type to incoming PFD measured in a large canopy gap. Incoming PFD was measured using a quantum sensor (LI-190SB, Licor, USA) mounted at 1.5 m above ground in the canopy gap where there was no vegetation to obscure the sky to within 52° of the zenith. Two additional quantum sensors were used to measure PFD in the invaded and native grass plots. We quantified sensor bias prior to measurements by placing sensors side by side and simultaneously logging PFD for four hours. From this comparison, a regression equation was derived and used to remove sensor bias. PFD sensors were then located 10 cm above ground, with measurements made every 1 m along three replicate 20 m transects within each plot-pairs at each of the four sites. Measurements from both sensors were simultaneously logged every 1 minute and converted into a percentage of incoming radiation reaching ground level. To reduce variation resulting from sun angle, measurements were made between 1030 and 1330 hours local time, with one plot-pair being measured per day. To further minimise bias due to sun angle, measurements alternated between treatment plots. Measurements were taken under direct and diffuse radiation conditions, however, measurements were not taken if conditions changed from direct to diffuse or vice versa during a measurement run.

We measured air temperature and humidity using shielded and ventilated temperature and relative humidity probes (CS 500, Campbell Scientific, Nebraska, USA) with measurements logged (CR10X, Campbell Scientific, Nebraska USA). Measurements were taken over a four-day period with a plot-pair measured for 22 hours per day (0900 to 0700 h the following day). For each grass canopy type, we installed a CS500 probe at 40 cm above the ground, supplemented by a further four shielded thermocouples (type T, copper-constantan) randomly located within a 20-m radius within the plot. Plot-pairs were measured simultaneously, with the sensor array located at four random locations within each treatment plot. VPD was calculated from T_air_ and RH% measures (after^51^. All sensors were logged using 30 minute averages, with sensor channels on the logger scanned every 10 seconds. To avoid biased sampling, consecutive days with contrasting meteorological conditions were avoided. Sensor bias was checked prior to fieldwork. Data summaries were prepared using the 30 minute means for each sensor, which were further averaged over the following local time periods: daytime mean (0900–1900 h); nocturnal mean (1900–0700 h); midday mean (1100–1500 h). The maximum values of each sensor were also logged. In February, T_air_ and PFD beneath the grass canopies was also measured simultaneously across all plot-pairs diurnally (0800–1730h), with mean values logged every 5 minutes. Sensors were mounted as described above within each canopy type.

Volumetric soil moisture (θ_v_) was measured in November, February, and April using a hand-held TDR probe (Theta Probe, ML2, Delta-T Devices, Cambridge, UK) with the probe inserted vertically into the soil to a depth of 6 cm. Measurements were taken every three metres along two ×24 m transects randomly located within each plot type. The mean value of each transect was used for statistical analysis. Measurements for all plot-pairs were collected on the same day.

### Seedling photosynthetic potential

We used the PFD and T_air_ data collected from each grass type to estimate the potential woody seedling assimilation (A_pot_) based on previously established relationships between *Eucalyptus* seedling photosynthesis and light and temperature^31^. In our scenario, A_pot_ represents the assimilation potential of a regenerating seedling for the temperature and light regime quantified for both native and gamba grass canopies. Prior *et al*.^31^ examined leaf scale photosynthetic properties of *E*. *tetrodonta* saplings at similar savanna sites in the Darwin region and developed relationships between leaf scale photosynthesis (A) and PFD and leaf temperature. Our temperature measure was air temperature and we assumed T_air_ and leaf temperature were similar during the wet season when leaf water stress and resultant elevated leaf temperatures would be at a minimum. These relationships were used to estimate A_pot_ during peak growing season conditions (February) using diurnal PFD and T_air_ data collected from each plot-pair at each site. Instantaneous values of A_pot_ were estimated and these were summed to give A_pot_ as g C per m^2^ d^−1^ for seedlings within each grass canopy type.

### Seedling establishment and survivorship

To investigate the effects of *A*. *gayanus* invasion on woody recruitment, we conducted a seed augmentation experiment using *E*. *miniata* and *E*. *tetrodonta* seed. Seed fall for these species occurs from September to December^52^ and seeds have high viability, but no innate dormancy and germinate quickly^30^. Seed of both species were collected near the study sites. This experiment was undertaken within the three plot-pairs with the highest cover of *A*. *gayanus*. Within each plot-pair, nine permanent 1 × 1 m quadrats were randomly located; three were sown with 250 *E*. *miniata* seeds, three with 250 *E*. *tetrodonta* seeds and three were “control” quadrats with no seed added. Quadrats were constructed using 5 cm steel mesh wrapped in flywire (after^30^ to stop seeds being washed out of the quadrats by the monsoonal rains that can result in significant runoff. In December, during the time of natural seed fall, seed (250 seed of either *E*. *miniata* or *E*. *tetrodonta)* was mixed with a handful of dry sand and sprinkled evenly across the quadrats, resulting in three replicate quadrats per species in each plot combination. The control quadrats enabled assessment of natural recruitment rates. Seedlings were counted fortnightly during the wet season from mid-January to mid-April, with a final count taking place in the dry season in mid-July. To determine whether differences in seedling recruitment between treatments could have been caused by seed predation, experiments took place in mid-November and mid-December. Seed depots consisted of ten *E*. *miniata* seeds placed in a 3–4–3 grid, with 10 cm spacing. This grid was repeated three times within a plot. Seeds were placed on a sprinkling of white sand to aid relocation. Seeds were counted daily for 2 days after^30^.

Seedling growth was assessed by tagging 20 randomly selected individuals per quadrat (or less if fewer than 20 individuals emerged) with numbered skewers. The height and number of true leaves were recorded in March for each tagged individual. Height was measured to the apical meristem and the presence of true leaves was confirmed if the pair of leaves had separated. Each seedling was re-measured four months later (May) to determine wet-season seedling growth rate. Growth of *E*. *miniata* seedlings was further quantified by measuring aboveground biomass and lignotuber biomass of five randomly selected seedlings quadrat, harvested in May and dried at 70 °C for 48 h (n = 90). To test for responses to light quantity, specific leaf area (SLA, leaf area/leaf dry weight) was calculated by measuring the length and width of a leaf at the third node from the apical meristem from all tagged seedlings to give an average leaf area per quadrat. Leaf weight was calculated by weighing the dried leaves from the harvested seedlings. Finally, to investigate any response to light quality (red:far-red ratio), internode length was compared by measuring the internode at the midpoint of each tagged seedling’s stem, and the foliage:stem weight ratio of dried seedlings was calculated to assess whether there was a difference in above-ground mass allocation. These data were not taken for *E*. *tetrodonta* because there were insufficient seedlings to undertake both harvest and survival monitoring. Seed predation and seedling survival were converted to proportion relative to the initial population.

Ground cover was recorded monthly between January and May, and the following July with % cover of grass, forbs, litter, woody re-shoots and bare ground recorded along with mean grass tussock height. Overstorey tree cover was recorded at the time of seed sowing and also at both seedling-growth measurement dates.

### Natural seeding recruitment

Natural seedling recruitment was assessed at seven random sites. At each site, six *E*. *miniata* trees surrounded by native grass, and six surrounded by *A*. *gayanus* were chosen. Trees were mature and at least 30 cm DBH and for each tree a circular plot was established with a radius of five (area = 78.5 m^2^) and seedling density within the plot measured. This is the zone of highest seed fall for these dominant *Eucalyptus* species^52^. Seedlings were identified as less than one year old based on the development of their lignotuber, which requires twelve to eighteen months to develop.

### Statistical analysis

The differences between the microclimate, environmental variables and A_pot_ in invaded and native grass plots at each sampling period were tested using two-factor mixed-model ANOVA’s, with factors grass canopy (native, gamba; fixed) and plot-pair (random). Seedling survival data was analysed with a three-factor ANOVA, with the factors including plot-pair (fixed), grass type (fixed) and species (fixed). Seedling biomass and seed predation data were analysed using a two-factor fixed analysis. Effect of gamba cover on natural seedling recruitment was analysed using a one-way ANOVA. Data that did not meet assumptions were either log-transformed, or arc-sine transformed for proportional data (seedling and seed predation data) to meet the assumptions of normality. *Post-hoc* pooling of treatment data was done if the plot-pair by treatment interaction was non-significant with P > 0.25^53^. Pearson correlation coefficients were used to investigate relationships between microclimatic factors, seedling recruitment and environmental variables.
